# Antithrombin III Deficiency in Indian Patients with Deep Vein Thrombosis: Identification of First India Based AT Variants Including a Novel Point Mutation (T280A) that Leads to Aggregation

**DOI:** 10.1371/journal.pone.0121889

**Published:** 2015-03-26

**Authors:** Teena Bhakuni, Amit Sharma, Qudsia Rashid, Charu Kapil, Renu Saxena, Manoranjan Mahapatra, Mohamad Aman Jairajpuri

**Affiliations:** 1 Protein Conformation and Enzymology lab, Department of Biosciences, Jamia Millia Islamia, New Delhi, India; 2 Department of Haematology, All India Institute of Medical Sciences, New Delhi, India; University of Bonn, Institut of experimental hematology and transfusion medicine, GERMANY

## Abstract

Antithrombin III (AT) is the main inhibitor of blood coagulation proteases like thrombin and factor Xa. In this study we report the identification and characterization of several variants of AT for the first time in Indian population. We screened 1950 deep vein thrombosis (DVT) patients for AT activity and antigen levels. DNA sequencing was further carried out in patients with low AT activity and/or antigen levels to identify variations in the AT gene. Two families, one with type I and the other with type II AT deficiency were identified. Three members of family I showed an increase in the coagulation rates and recurrent thrombosis in this family was solely attributed to the rs2227589 polymorphism. Four members of family II spanning two generations had normal antigen levels and decreased AT activity. A novel single nucleotide insertion, g.13362_13363insA in this family in addition to g.2603T>C (p.R47C) mutation were identified. AT purified from patient’s plasma on hi-trap heparin column showed a marked decrease in heparin affinity and thrombin inhibition rates. Western blot analysis showed the presence of aggregated AT. We also report a novel point mutation at position g.7549 A>G (p.T280A), that is highly conserved in serpin family. Variant protein isolated from patient plasma indicated loss of regulatory function due to *in-vivo* polymerization. In conclusion this is the first report of AT mutations in SERPINC1 gene in Indo-Aryan population where a novel point mutation p.T280A and a novel single nucleotide insertion g.13362_13363insA are reported in addition to known variants like p.R47C, p.C4-X and polymorphisms of rs2227598, *Pst*I and *Dde*I.

## Introduction

Thrombosis is a complex disease associated with most of the vascular disorders including myocardial infraction, cerebrovascular & peripheral arterial diseases and deep vein thrombosis (DVT). Under normal conditions, procoagulant, anticoagulant and fibrinolytic pathways regulate hemostasis to avoid pathological clot formation [1, 2]. However, any inadvertent clotting either due to mutation of procoagulant or anticoagulant factors or due to acquired factors may lead to life threatening thromboembolic disorders. Inherited causes of thrombosis include mutations in the genes that encodes for AT, protein C (PC), protein S (PS), factor V and factor II [3].

Antithrombin III (AT), a member of serpin (serine proteinase inhibitor) super-family is the most important endogenous anticoagulant. The significance of AT in hemostasis is evident by the fact that its heterozygous deficiency is associated with increased risk of thrombosis whereas homozygous deficiency might be fatal. AT regulates coagulation by inhibiting thrombin, factors IX, Xa and XI of the blood coagulation system. AT has evolved a complex heparin induced conformational change mechanism to efficiently inhibit these proteases. However this has also made AT prone to structural and functional defects [4]. AT gene (SERPINC1) spans 13.4kb of genomic DNA and is located on chromosome 1q23-25 [5]. The first mutation linked to AT deficiency was characterized in 1983 [6]. AT deficiency may be either type I, where both the activity and antigen levels in the plasma are reduced or type II, where normal antigen levels are associated with a reduced AT activity level. Variants like AT Murcia (K241E) causing altered glycosylation pattern [7], AT Rouen-IV & AT London (R24C) [8, 9] that leads to abolition and reduction in inhibitory activity, or those like F229L [10] causing spontaneous *in vivo* polymerization have provided valuable insight into the underlying mechanism of AT deficiency. In addition to point mutations, rs2227589 polymorphism located at 140bp downstream of exon 1 in the AT gene has also been shown to be associated with a high risk of thrombosis. In a study conducted by Bezemer et al. on 19682 SNPs located in 10887 genes, rs2227589 was found to be one of the three polymorphisms associated with a high risk of DVT [11].

Venous thromboembolism (VTE) for long has been perceived to be less common in Asian population compared to western population [12]. However, population-based epidemiological study in Asian countries has demonstrated an increasing incidence of VTE in Asians annually [12] and mutations of AT gene in Asian population has been reported [13–15]. Although the first report of AT deficiency in an Indian family dates back to 1982 [16], yet no mutation has been identified in Indian population till date.

In the present study we report the first ever analysis of Indian population with DVT for AT deficiency. Mutations and additional thrombotic risk factors in AT gene were studied in 52 unrelated Indian patients with low AT levels. We report two Indian families, one with type I and the other with type II AT deficiency and the genetic defect in AT gene underlying thrombosis within these families and other unrelated individuals. A novel single nucleotide insertion at g.13362_13363insA and a novel point mutation at position g.7549 G>A causing p.T280A substitution were identified as the genetic basis of DVT. Further analysis of the AT variants showed presence of polymerized AT in patients with type II AT deficiency which leads to reduction in the level of AT activity.

## Methods

### Patients

We screened 1950 Doppler-proven DVT patients in the time span of about two years (October 2011-August 2013) for AT deficiency. Only patients with AT based thrombosis were included in the study and patients with surgery, accident, trauma, pregnancy, central venous catheter, malignancy, infection, dehydration, immobility, systemic illness or low anticoagulant levels other than AT were excluded. Family studies were carried out wherever available. All the patients gave written consent to enter the study and ethical clearance was obtained from Institute Ethics Committee of All India Institute of Medical Sciences (AIIMS) and Jamia Millia Islamia University, New Delhi, India.

### AT assay and other thrombophilic tests

Plasma AT activity levels were determined by amidolytic heparin cofactor assay with chromogenic substrate CBS 61.50 (STA-STACHROM ATIII; Diagnostica stago, France). AT antigen levels were determined by latex immunoassay (LIATEST ATIII; Diagnostica stago, Asnieres, France). Prothrombin time (PT), thromboplastin time (TT) and activated partial thromboplastin time (APTT) were performed using kits from Diagnostiga STAGO. Plasma levels of PC and PS were measured by enzyme-linked immunosorbent assay and the kits used were Asserachrom sEPCR ELISA from Diagnostica Stago (Asnières, France). Homocysteine (SHO) and Beta-2 glycoprotein (β-2 gp) levels were measured by enzyme immunoassay by using kits from AXIS-SHIELD, Scotland and GA Generic assays GmbH, Germany respectively. All the assays were carried out as per the manufacturer’s protocol.

### Genomic Sequence analysis

RNA-free genomic DNA was isolated from 5 ml of venous blood using the Bioserve DNA isolation kit. DNA concentration was determined using Eppendorf BioPhotmeter plus NanoDrop device. Polymer chain reaction (PCR) for SERPINC1 gene variations was done with primer details and amplification conditions as described elsewhere [10, 17, 18] and in S1 Table. PCR products were purified from agarose gel using silica bead DNA gel extraction kit (FERMENTAS) and were subjected to DNA based sequencing using 96 capillary high throughput sequencer; ABI 3730 XL, with both forward and reverse primers used for amplification. Comparison with the reference sequence (GenBank accession number NG_012462.1) was performed with MEGA 6.0 software [19]. Allelic and genotypic frequencies and p values for the polymorphisms, *Pst*I and rs2227589 were determined using SNPstats software [20]. Statistical significance was taken as p<0.05. Data are presented as mean ± standard error (S2 Table).

### *In silico* analysis

Sequence information acquisition: 27 serpin inhibitory protein sequences including wild type AT were extracted from NCBI protein database [21]. Translated mutant AT sequence was obtained from gene sequencing experiment and was translated into protein using expasy translator tool [22].

### Structure prediction for g.13362_13363insA in family II

Since, mutated exon 6 bears poor homology with any known structure, so its tertiary structure was predicted using threading based method, I-TASSER [23]. Further, MODELLER [24] was used for the prediction of complete mutant AT bearing mutated exon 6. The template used in MODELLER was native AT (PDB ID: 1E05 chain I) and I-TASSER generated mutant exon 6. Multiple template based modeling was performed and structure validation was carried out in verify3D server [25].

### Sequence alignment, phylogeny and Exon 6 sequence conservation

Multiple sequence alignment (MSA) of 27 inhibitory *Homo sapien* serpins was carried in EBI-ClustalW using neighbor Joining Clustering method, with the Gap opening and extension penalties of 10 and 0.2 respectively and setting the output order as “input”. Exon 6 and AT strand 4B were extracted along with mutated exon 6 of our variant AT and the sequences were analyzed for conservation. WebLogo [26] server was used for the generation of conservation plot of exon 6.

### Computation of Accessible Surface Area (ASA) and free energy change

ASA of T280 residue of native AT (PDB: 1E05) was computed by ASA View [27]. I-mutant2.0 analysis was carried out at temperature 25°C and pH 7.0 to compute the free energy change upon point mutation [28].

### AT purification and electrophoretic analysis

Variant AT of proband of family II was purified from plasma (4ml) by heparin affinity chromatography using a 1ml Hi-trap column from ToyoScreen. Thrombin inhibitory activity of eluted fractions was checked using chromogenic substrate S-2238 (H-D-Phenylalanyl-L-pipecolyl-L-arginine-p-nitroaniline dihydrochloride) at 405nm. Protein concentration of purified AT was determined from absorbance at 280 nm using the molar extinction coefficient of plasma AT (0.66) [29]. Pooled fractions of p.T280A variant were passed through a gel filtration column (10cm×2.5cm, sephadex G-100) in 1X Phosphate-Sodium-EDTA (PNE) buffer, (pH = 7.4). The peaks obtained after gel filtration were pooled, concentrated and analyzed on SDS-PAGE and non-denaturing PAGE as described earlier [30].

### Western blotting

Proteins separated by gel electrophoresis were blotted to polyvinylidene fluoride membranes and AT was immune stained with polyclonal rabbit antihuman SERPINC1 antibody (Sigma, St Louis, MO, USA). This was followed by incubation of the membrane with polyclonal goat anti-rabbit IgG-alkaline phosphatase (Sigma, St Louis, MO, USA) with detection via BCIP/NBT tablets (Sigma, St Louis, MO, USA).

### Circular Dichroism (CD) analysis

The effect of temperature on AT secondary structure was monitored at 222nm [31] on an Applied Photophysics Circular Dichroism spectropolarimeter equipped with peltier-type temperature controller. A heating rate of 60°C/h was used and the scan was recorded from 30–90°C with a response time of 12 sec, bandwidth of 1nm & cuvette with 10mm pathlength, protein concentration was 2μM in 1XPNE (pH = 7.4). Melting temperature (T_m_) of wild type and mutant AT were calculated as described earlier [32]. Far-UV CD spectra (200–260nm) were recorded to determine the changes in secondary structure using a 10mm path length cell.

### Bis-ANS fluorescence

To 500nM of mutant protein, 2μl of 3.0mM Bis-8-anilino naphthalene 1-sulfonate (ANS) was added and the sample was excited at 390nm and emission spectra were recorded in 400-600nm range.

## Results

### Screening of DVT patients

Out of 1950 Doppler proven DVT patients assessed in this study, 10.76% (210) had low protein C and 8.7% (170) had low protein S levels. An AT based testing identified 2.66% (52) patients with low AT levels. Most of the patients with low AT antigen and/or activity levels were also found to have deranged coagulation time as assessed by APTT, PT and TT assays.

### Sequence analysis and clinical features of AT deficient patients

SERPINC1 gene of 52 patients with low AT levels were analysed by direct DNA based sequencing (Table 1). Two families and three individual cases were found to have genetic variations in the SERPINC1 gene.

**Table 1:** AT mutations, phenotypic and genotypic features identified in Indian DVT patients.

**Patient ID No.**	**Subject**	**Exon/ Intron**	**Change Identified**	**Type of AT deficiency**	**APTT/ PT/TT (seconds)** ^1^	**Age of first thrombosis/ Sex**	**Clinical features**	**AT activity (%)**	**AT antigen (%)**
FI	I:2		786G	I	12.33, 5.00, 14.00	NA/M	Asymptomatic	55	40
	II:1	Intron 1	786A	I	8.00, 6.00, 18.33	20/M	RecurrentDVT	52	40
	II:2	Intron 1	786A	I	12.33, 7.00, 15.00	19/M	RecurrentDVT	55	39
FII	I:2	Exon 2	2603C> T	II	7.50, 10.00, 19.00	NA /F	Asymptomatic	28	100
	II:1	Exon 2, 6	2603T> C, 13363insA	II	6.33, 10.00, 20.00	28/F	DVT	57	94
	II:5	Exon 2, 6	2603T> C, 13363insA	II	9.00, 11.00, 8.00	NA /F	Asymptomatic	75	100
	II:6	Exon 2, 6	2603T> C, 13363insA	II	15.66, 7.00, 18.50	NA /M	Asymptomatic	68	100
PRS	-	Exon 2	2455C>A	I	14.00, 12.00, 8.00	52/M	DVT	65	60
PSh	-	Intron 5	9893 G>C	I	9.00, 6.00, 8.00	12/M	DVT	69	50
PRn	-	Exon 4	7549 A>G	II	9.00, 13.00, 15.50	30/F	DVT	53	96
PRa	-	Exon 4	7626A>G	I	6.00, 6.00, 15.50	35/F	Recurrent DVT	75	52
PSBe	-	Exon 4, Intron 1	7626G>A 786G>A	I	7.00, 4.00, 11.33	23/F	RecurrentDVT	6	65
PSa	-	Exon 4, Intron 1	7626G>A 786G>A	II	6.30, 7.00, 12.00	24/F	Recurrent DVT	77	83
PSB	-	Exon 4	7626A>G	I	10.00, 7.00, 10.00	34/M	ACD^2^ with chronic BCS^3^	77	75
PNJ	-	Exon 4	7626A	I	9.50, 13.00, 8.00	26/F	Recurrent DVT	65	79

^1^ Normal laboratory ranges: APTT; 25s, PT; 13s and TT; 16s

^2^ Acute Coronary Disease

^3^ Budd Chiari Syndrome

#### Family I

**Fig 1:**
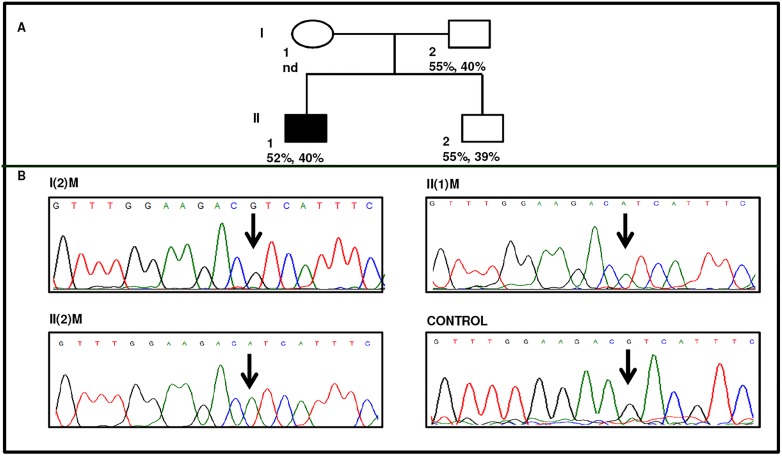
Identification of AT deficiency and rs2227589 in family I. A) Pedigree of proband. Proband is indicated by dark square. Anti-fXa activity and antigen levels are indicated in the second row respectively. nd indicates not determined. B) Electropherogram indicating that proband, II(1)M and his sibling II(2)M are homozygous for AA at position 786 and father, I(2)M is homozygous for wild type GG at the indicated position.

Three patients in family I were with very low and comparable AT activity and antigen levels (Fig. 1A and Table 1). The proband in family I had a stroke at the age of 18 years and his younger brother was also suffering from thrombosis. Anticoagulant based testing revealed significantly low AT levels in all three. Further, considerably reduced PT indicated a hypercoagulable state due to defects in extrinsic pathway (Table 1). Nucleotide sequencing of the entire protein encoding region of the SERPINC1 gene revealed no changes. Complete sequencing of the introns and promoter region showed variation at position 786 (rs2227589) in the symptomatic proband and his sibling but not in asymptomatic father. The known polymorphism [17] was confirmed by sequencing of the intron I region from both forward and reverse primers, and sequencing of the region containing the polymorphism was repeated three times with fresh template each time (Fig. 1B). In addition to family I, rs2227589 polymorphism was also studied in rest of the DVT patients and the allele frequencies were estimated to be G:0.86 and A:0.14 (S2 Table).

#### Family II

**Fig 2:**
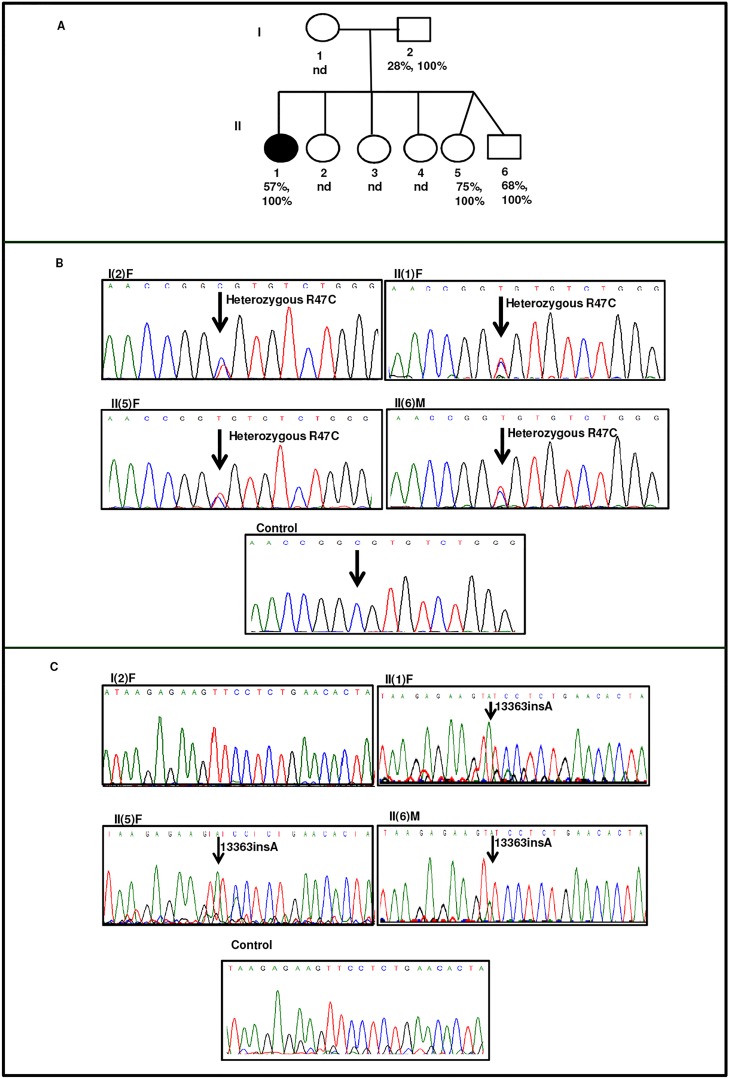
Identification of AT deficiency and mutation in family II. A) Pedigree of family II. The proband, II(1)F are represented by dark oval. Anti-fXa activity and antigen levels are shown in the second and third row respectively. nd indicates not determined. B) Electropherograms indicating that proband, II(1)F and family members are heterozygous for 2603T>C R47C mutation. C) Electropherogram of the proband and family members are homozygous for insertion of A at position 13363. The electropherograms of family members is compared with that of the healthy control. The insertion of A causes a frameshift that changes the open reading frame.

In family II, mother and three of the six siblings in the second generation were presented with low AT activity levels and normal antigen levels (Table 1). The proband II(1)F was a 28 year old Indian female who had recurrent abortions in August 2009, January 2010 and January 2012 and was referred to AIIMS. PC (100%), PS (90%), β-2 gp (1%) and SHO (14μmol/l) levels were found to be normal, while significant reduction was found in AT activity levels with normal antigen levels (57%, 94% respectively). APTT levels were found to be drastically reduced with normal PT and TT levels (Table 1). Blood samples were obtained from family members where available and sequencing of the entire exonic region of SERPINC1 gene was carried out. A previously reported point mutation at position g.2603T>C (p.R47C) [33] was observed in four members of this family (Fig. 2B). It has been reported previously that R47 located in the helix-A is involved in interaction with heparin, and the variant (R47C) is known to compromise its heparin binding affinity [34]. In addition to R47C variant, we also observed a single nucleotide insertion, g.13362_13363insA in the proband and siblings. This insertion was however absent in the mother (Fig. 2C).

#### Case Report I

In another patient (PRS), presented with type I AT deficiency an unpublished point mutation leading to premature termination of the protein (p.C4-X) [33] was detected in exon 2 at position g.2455C>A (S1 Fig.). The patient was suffering from DVT with vascular headache and vertigo. PC (110%), PS (90%), β-2 gp (1%) and SHO (14μmol/l) levels were normal with reduction in AT activity and antigen levels (65%, 60% respectively). Family members of the proband were not available for the study, and this mutation was absent in rest of the patients.

#### Case Report II

In a 12 year old boy suffering from DVT, a previously known *Dde*I polymorphism was identified at position g.9893 G>C [33] (S2 Fig.). Anticoagulant based testing identified reduced AT activity and antigen levels (69%, 50% respectively) whereas PC, PS, β-2 gp and SHO levels were normal (100%, 110%, 3%, 12 μmol/l respectively). The polymorphism was checked in rest of the DVT patients & control and was found to be absent.

#### Case Report III

**Fig 3:**
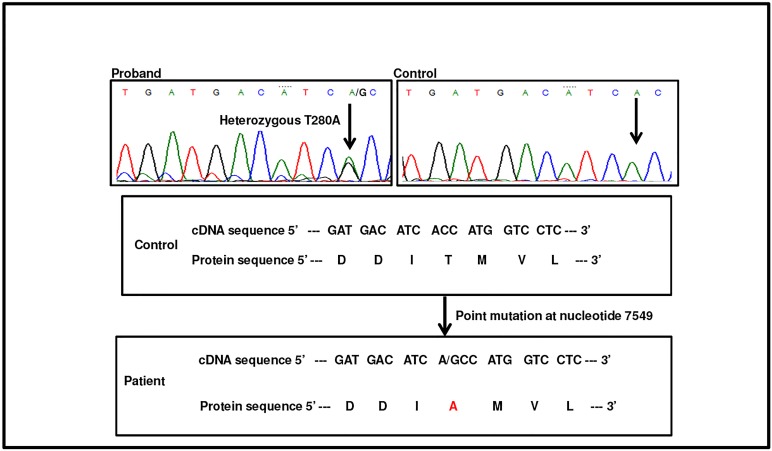
Identification of T280A mutation. Electropherograms indicating heterozygous 7459 A>G (T280A) mutation in patient compared with healthy control.

A novel point mutation at position g.7549 A>G was observed in an Indian female DVT patient (PRn) belonging to a remote area of the country. PC (80%), PS (90%), APCR (120 seconds), β-2 gp (1%) levels were normal, while significant reduction was found in AT activity level (53%) with normal antigen level (96%). APTT was significantly reduced whereas PT and TT levels were normal (Table 1). The heterozygous mutation (g.7549 A>G) results in the substitution of alanine in place of threonine (p.T280A). Electropherogram indicating the mutation is shown in Fig. 3. Family members were not available for the study.

In addition *Pst*I was also identified in some patients with low AT levels and had allele frequencies of G:0.5, A:0.5. The frequencies are listed in S2 Table.

### Biochemical characterization of compound heterozygote (p.R47C, g.13362_13363insA) in family II

**Fig 4:**
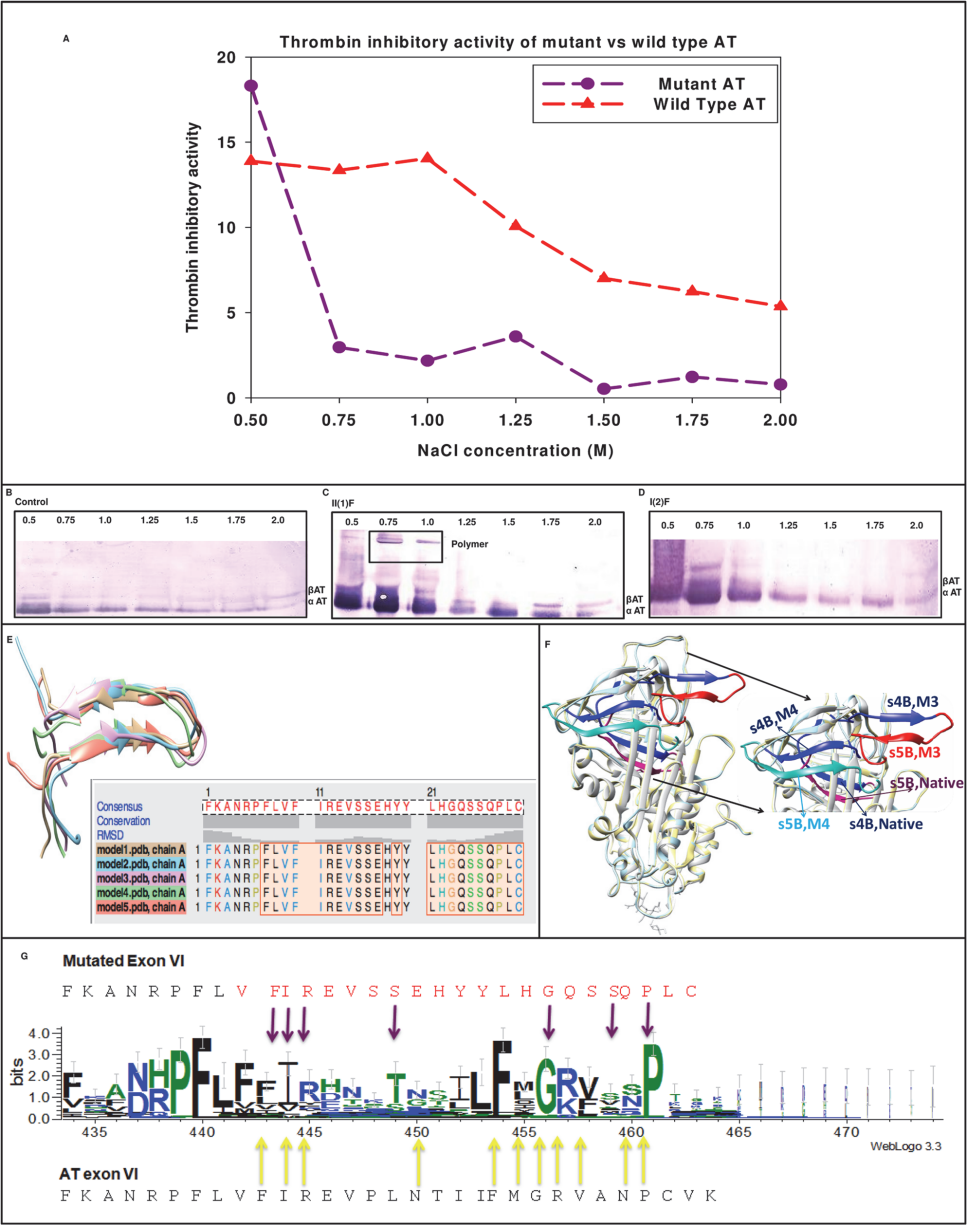
Characterization of compound heterozygote (2603 T>C, ins13363A) in family II. A) Thrombin inhibitory activity of mutant AT compared with wild type AT. The results plotted are average of three independent experiments. B) SDS-PAGE followed by immunoblotting in healthy control. C) SDS-PAGE followed by immunoblotting in the proband, II(1)F. Pure AT at 2.0M NaCl concentration serves as an internal control as α and β isoforms of AT elutes at equimolar concentration. D) SDS-PAGE followed by immunoblotting in the mother of proband, I(2)F. E) Superimposition of 5 predicted models of N-terminal 30 residue stretch resultant of frame shift mutation F) Tertiary structure of mutated AT with 13363 insA. Hot pink represents 1E05:1 (wild type), red indicates model 3 and teal represents model 4. Blue denotes common among these structures. G) WebLogo of 27 inhibitory serpins including mutant exon 6. Molecular graphic images were prepared in Chimera.

The variant AT in proband of family II had two point mutations (Fig. 2). Thrombin inhibitory activity of eluted fractions from Hi-trap heparin column showed decreased activity of the variant (Fig. 4A). Western blot showed reduction in the heparin binding affinity as indicated by elution of protein at lower ionic strength (Fig. 4B). It showed the presence of an abnormal complex at higher molecular weight, whereas this band was absent in the mother indicating that mutation (g.13362_13363insA) around the s4B and s5B in AT is the probable reason for this band and an indication of oligomerization of AT variant that compromises its inhibitory activity.

The protein sequence translated from the AT gene sequence of proband revealed a frameshift mutation p.P416SfsX16 that results in the formation of a new C-terminal coding sequence, causing early termination. 22 out of 30 C-terminal residues in mutated exon 6 coded for an entirely new protein sequence. Tertiary structure of mutated AT modelled structure in comparison with the wild type AT revealed a contrasting conformational change in P416-C431 stretch that is coded by mutated exon 6. We observed that residues P416-K432 which are deep seated in wild type become exposed in case of the mutant AT (p.P416SfsX16). Possible orientation of this stretch showed that expulsion of s3B & s4B may have contributed to oligomer formation by loop-sheet mechanism (Fig. 4F). WebLogo analysis of wild type exon 6 (V375-K432) of 27 serpins with mutated exon 6 of variant AT listed few conserved residues that were sustained post mutation (Fig. 4G).

### Biochemical characterization of p.T280A (case report III)

**Fig 5:**
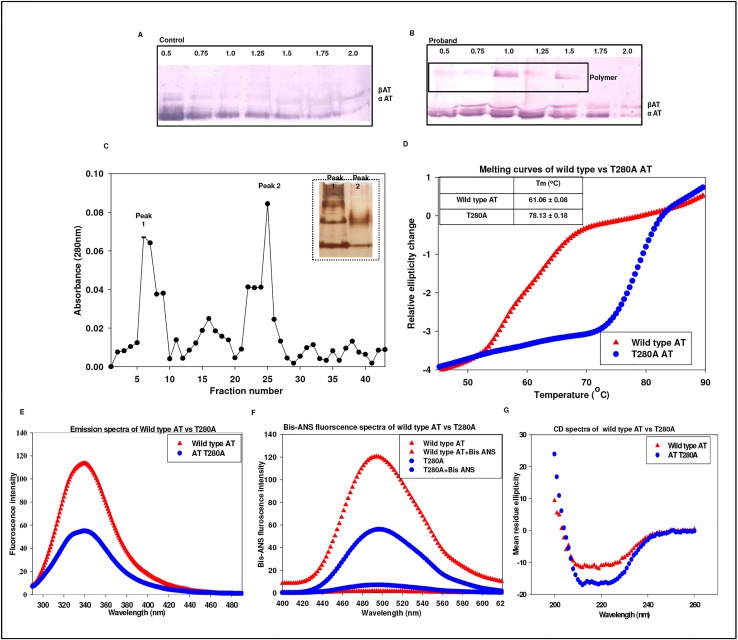
Biochemical characterization of T280A. A) SDS-PAGE followed by immunoblotting in healthy control. B) SDS-PAGE followed by immunoblotting in proband, indicating presence of high molecular weight AT species in proband’s plasma. Pure AT at 2.0M NaCl concentration serves as an internal control as α and β isoforms of AT elutes at equimolar concentration. C) Elution profile of the polymerized and native species using gel filtration chromatography (G-100). Peak 1 and peak 2 are polymerized AT containing a low level of native AT. Shown in inset is NATIVE-PAGE indicating that T280A mutation causes spontaneous polymerization of AT in patient’s plasma. Lane 1: peak 1, lane 2: peak 2. D) Thermal stability of T280A and wild type AT. Mean ± S.D are reported for measurements done in duplicate. E) Fluorescence emission spectra of wild type compared with T280A AT. F) Wild type and T280A AT hydrophobicity studies by Bis-ANS fluorescence studies. G) Far UV-CD spectra of wild type and T280A AT showing changes in secondary structure. The results plotted are average of atleast three independent experiments.

**Fig 6:**
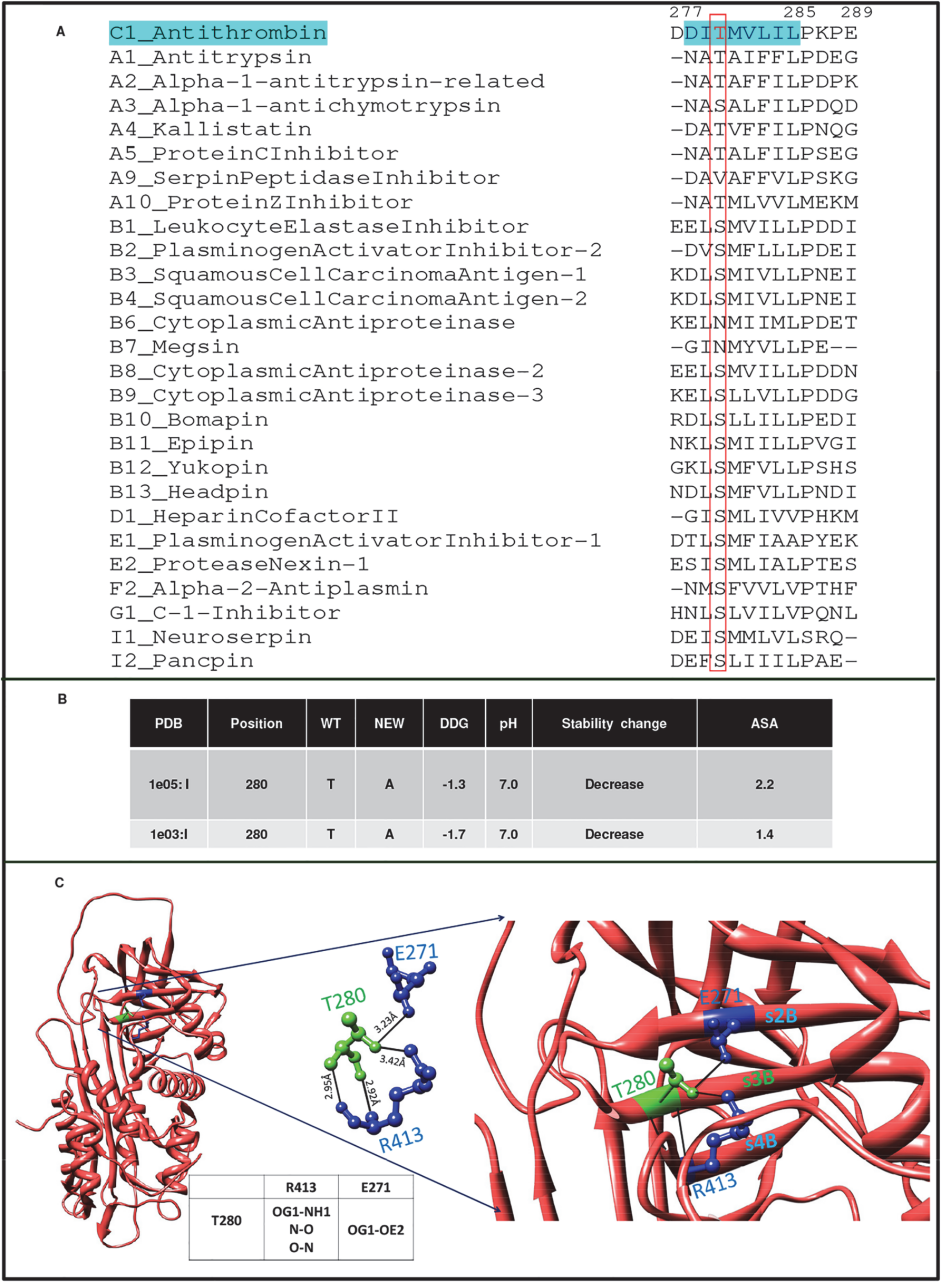
*In silico* characterization of T280A. A) Multiple sequence alignment of strand 3 sheet B (s3B) of AT with 27 *Homo sapien* inhibitory serpins. B) ASA of T280; PDB:1E05, I mutant analysis to study change in energetics and stability in T280A. C) T280 Hydrogen bond interactions in native AT (PDB:1E05). Molecular graphic images were prepared in Chimera.

The mutant p.T280A AT resulting from g.7549G>A substitution was purified using heparin affinity chromatography, Western blot showed the presence of high molecular band (polymer like) in the proband (Fig. 5B). Fractions with polymer like band were pooled & buffer exchanged and run on sephadex G-100 gel filtration column (Fig. 5C) and two major peaks, designated as peak 1 and peak 2 were obtained. The purified fractions of peak 1 and peak 2 were assessed for polymer formation on the NATIVE-PAGE using silver staining (Fig. 5C inset). NATIVE-PAGE showed the presence of high molecular weight polymers of T280A in peak 1. Fractions of Peak 1 containing high molecular weight bands were pooled, concentrated and subjected to structural analysis (Fig. 5D-5G). A temperature dependent analysis showed that T_m_ (78.13 ± 0.18) of this pooled fraction presumed to contain p.T280A mutant was much higher than the wild type AT (61.06.45 ± 0.08), where the T_m_ of wild type was similar as reported earlier (Fig. 5D) [35]. Fluorescence emission spectra showed a significant decrease in emission intensity at 340 nm as compared to the wild type indicating shielding of tryptophans in the variant and different tertiary structure (Fig. 5E). Bis-ANS binding to p.T280A (Fig. 5F) showed a comprehensive reduction in the surface exposed hydrophobic patches in the variant as compared to the wild type AT. CD spectra of the variant showed that p.T280A has increased α-helical content and decreased β-strands (Fig. 5G). Position T280 in serpin-superfamily is conserved (Fig. 6A) and ASA analysis showed this threonine to be deeply buried, where T280A variant may destabilize the protein (Fig. 6B). T280 hydrogen bonding to E271 and R413 in the native state (Fig. 6C) may be leading to its destabilization in the variant.

## Discussion

Inherited AT deficiency has long been associated with DVT. In addition to more than 250 mutations that are known in the AT gene rs2227589 polymorphism has also been consistently shown to be associated with slightly lower levels of AT activity and plasma antigen levels [17]. A Dutch study identified rs2227589 along with two other polymorphisms as strong genetic factors contributing to DVT. It was performed on 3 large case control studies, LETS (443 cases and 453 controls; OR: 1.42; p-value: 0.03), MEGA-1 (1398 cases and 1757 controls; OR: 1.24; p-value: 0.01) and MEGA-2 (1314 cases and 2877 controls; OR: 1.29; p-value: <0.001), and indicated that rs2227589 was associated with a modest thrombotic tendency [11]. A meta-analysis performed later on 1076 cases and 1239 controls in black population with an aim of replicating the Dutch study confirmed association of rs2227589 with DVT [36].

**Fig 7:**
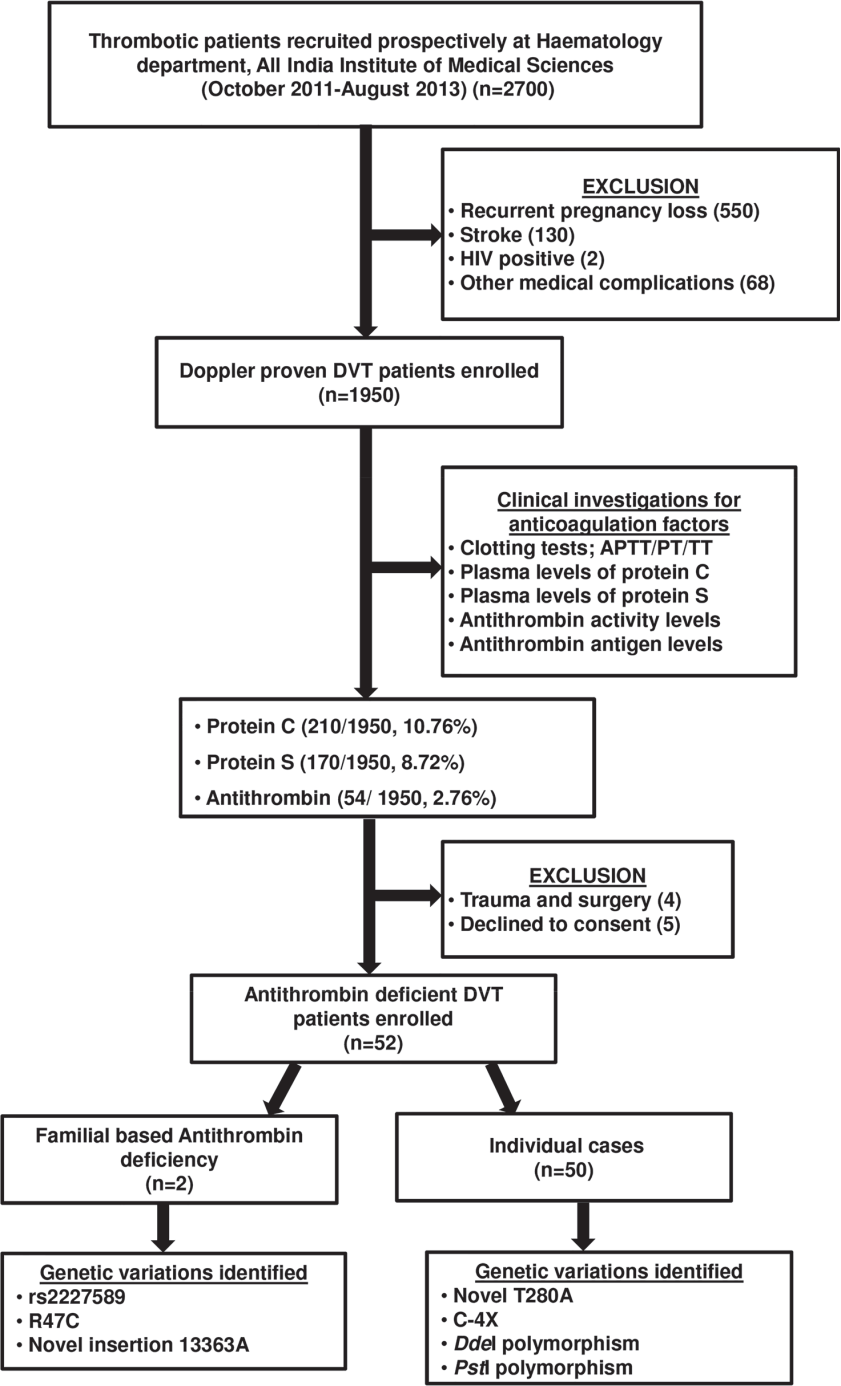
Flow chart summarizing the patients recruited, techniques employed and results of the study.

In the present study, two families with different defects in AT gene leading to DVT have been identified in Indian population. A flow chart summarizing the features of the study is presented as Fig. 7. The underlying cause of DVT in family I with low AT antigen and activity levels along with drastically reduced PT was found to be rs2227589. The presence of homozygous AA genotype in proband and his younger brother explains the probable cause of DVT. The presence of rs2227589 in absence of any other mutation in protein coding region makes it important to study this polymorphism in cases where no acquired or genetic factor appears to be contributing to DVT. In addition to family I, rs2227589 was also found to be present in other DVT patients with lower AT levels. The frequency of A allele (0.14) in Indian patients was found to be consistent with that reported earlier in Caucasian population, A: 0.12 (The Dutch study) and indeed AA genotype appeared to be associated with lower AT levels and was not found in any of the healthy controls (p value: <0.0001) (S2 Table). Furthermore, *Pst*I polymorphism was observed in family II and other patients but was found to be only a genetic variation in SERPINC1 gene as reported earlier [37] and was not associated with lower AT levels (p value: 0.4) (S2 Table).

Type II AT deficiency is known to affect either the reactive site domain, heparin binding site (HBS) or have pleiotropic effects. These changes are caused by single base pair substitutions and only two exceptions are known, where a deletion or insertion has been reported to be associated with type II AT deficiency. One such variant is AT London, which lacks R393 resulting in loss of inhibitory activity [9] and the other is a 24 nucleotide insertion reported in a 51-year old Caucasian male located between strand 3A (s3A) and helix F [38] that caused severe thrombotic history in the family. In our study the proband in family II was presented with a hypercoagulable state as indicated by reduced APTT and low AT activity levels. The family members were diagnosed with a type II AT deficiency and were found to have a single nucleotide insertion, g.13362_13363insA and a point mutation causing p.R47C substitution (Table 1, Figs. 2 & 4). The single insertion g.13362_13363insA was present in three members of the family and resides near s4B and s5B of AT whereas g.2603T>C (p.R47C) mutation present in all the four members resides in the HBS of AT. The single base insertion causes frameshift of amino acids from 416 to 432 (p.P416SfsX16).

Elution profile (Fig. 4A) showed elution of variant AT at lower ionic strength from Hi-trap heparin column as indicated by shift in the inhibitory activity and appearance of larger peak at much lower ionic strength. Oligomeric bands were observed in the Western blot of the proband but not in the mother who carried only p.R47C variant. This clearly indicates that it is the conformational change associated with insertion g.13362_13363insA which increases the polymer forming ability of variant and p.R47C decreases the heparin binding affinity of variant AT. Previous studies also indicate that mutations in this region lead to a global conformational change abolishing thrombin inhibition and decreasing heparin affinity [39]. Mutations affecting this region relay structural changes in the distal HBS by perturbing the B sheet and the core of the molecule [40] and both these findings are supported by our study. Exposure of the mutated region as opposed to deep seated structure in wild type AT as observed by *in silico* study explains the probable mechanism of polymerization. The frame shifted region was modelled beyond residue number 416 using a thread based structure modelling of the 22 C-terminal residues and indicated the maintenance of the sheets. Modelled structure showed expulsion of s3B and s4B (Fig. 4E). Expulsion of strands of β-sheet B may result in the insertion of RCL of another intact molecule into the sheet B resulting in loop sheet type of polymer formation.

T280 resides on strand 3B in a highly conserved region in serpin superfamily (Fig. 6A). Based on the biochemical and *in silico* studies we can predict the nature of the changes in the conformation of p.T280A as compared to the wild type AT. Indication of abnormal complex on Western blot analysis of AT purified from patient plasma (Fig. 5B) and massive increase in T_m_ (Fig. 5D) indicated high molecular weight polymer like species. Similar observations have been made previously where neuroserpin polymers formed *in vitro* were accompanied by massive increase in T_m_ [41]. A decrease in the fluorescence emission spectra (Fig. 5E), decrease in the overall exposed hydrophobic surfaces (Fig. 5F) and alteration in the secondary structure of the variant (Fig. 5G) as compared to the wild type indicated the presence of a structure which is quite different from the wild type and is compact like a polymer. T280 is deeply buried (Fig. 6B) and is involved in hydrogen bond interactions with R413 and E271 (Fig. 6C). One plausible mechanism for the presence of high molecular weight band in the Western blot may be that the removal of side chain hydrogen bond with β-strand 2B (E271) and β-strand 4B (R413) might have introduced local destabilization resulting in the opening of sheet B (Fig. 6B). This may allow insertion of RCL of intact AT into the β-sheet B producing loop sheet type of oligomers (Fig. 5C). An increase in the high molecular weight polymer bands was observed in the p.T280A variant (peak 1, Fig. 5C inset), run on size exclusion sephadex G-100 column. As previously reported, ATIII Budapest (P429L) and other natural variants in strand 1C (P407L) are in close proximity originating in β-sheet B, and are associated with considerable loss of heparin affinity and protease inhibition ability [39, 40]. Close proximity of this region with RCL influences thrombin binding and leads to loss of heparin affinity that indicates its long range connectivity with HBS. Further, a sheet B variant, G424R at the carboxy terminus has been reported to be associated with presence of polymer like abnormal complex [42]. Based on these collective evidences it seems that mutation at position g.7549 A>G (p.T280A) identified in our study causes polymerization of AT *in vivo*, thus resulting in clinical manifestation of disease in the patient with type II AT deficiency. Indication of polymers in G424R variant and in our study and the presence of serine/threonine in equivalent position in highly conserved region in most of the serpin family members (Fig. 6A) and observation of an increase in the α-helical content (Fig. 5G) in our study warrants more work to resolve the molecular basis of defects associated with this region.

In conclusion, AT as a risk factor for DVT in Indian population is not different from that reported in western population. This is the first Indian study where novel and known variants are identified in AT gene in DVT population. Two families and three individual patients with low AT levels and genetic variations in AT gene were identified. A known polymorphism previously known to be associated with thrombotic risk was found to be the underlying cause of DVT in one family and in another family two independent mutations causing conformational change in AT leading to thrombosis was identified. A novel point mutation (p.T280A) was identified in a highly conserved region and is hypothesized to be involved in polymerization. The detection of mutations and polymorphisms like C-4X, g.13362_13363insA, g.2603T>C (p.R47C), g.7549G>A (p.T280A), rs2227589, *Pst*I and *Dde*I in Indian population warrants a much larger study to understand prevalence and molecular basis in large Indian population (1.3 billion).

## Supporting Information

S1 Fig Electropherogram shows a heterozygous 2455 C>A (C4-Stop) mutation in the patient as compared with healthy control. (TIF)

S2 Fig Electropherogram shows a heterozygous 9893 G>C (*Dde*I polymorphism) mutation in the patient as compared with healthy control. (TIF)

S1 Table Primer details used for PCR amplification of SERPINC1 gene. (DOCX)

S2 Table Genotype frequencies and their association with plasma AT activity and antigen levels in patients and healthy controls. (DOCX)
