# Supplementary figures and images for: Antithrombin III Deficiency in Indian Patients with Deep Vein Thrombosis: Identification of First India Based AT Variants Including a Novel Point Mutation (T280A) that Leads to Aggregation
Source: PLoS One. 2015 Mar 26;10(3):e0121889. doi: 10.1371/journal.pone.0121889 (PMC4374914; doi:10.1371/journal.pone.0121889)

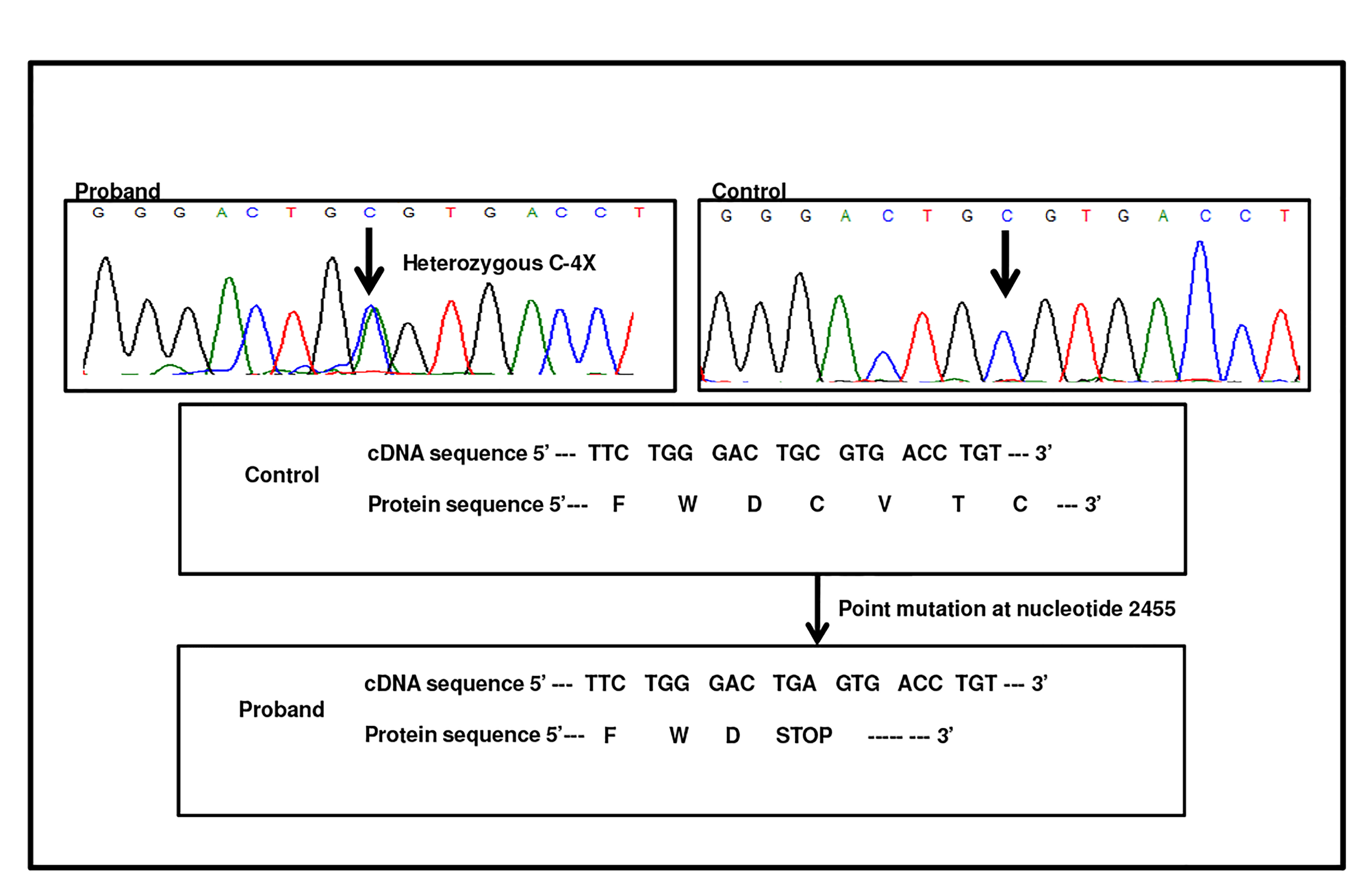

Supplement: S1 Fig — (TIF) [file pone.0121889.s001.tif]

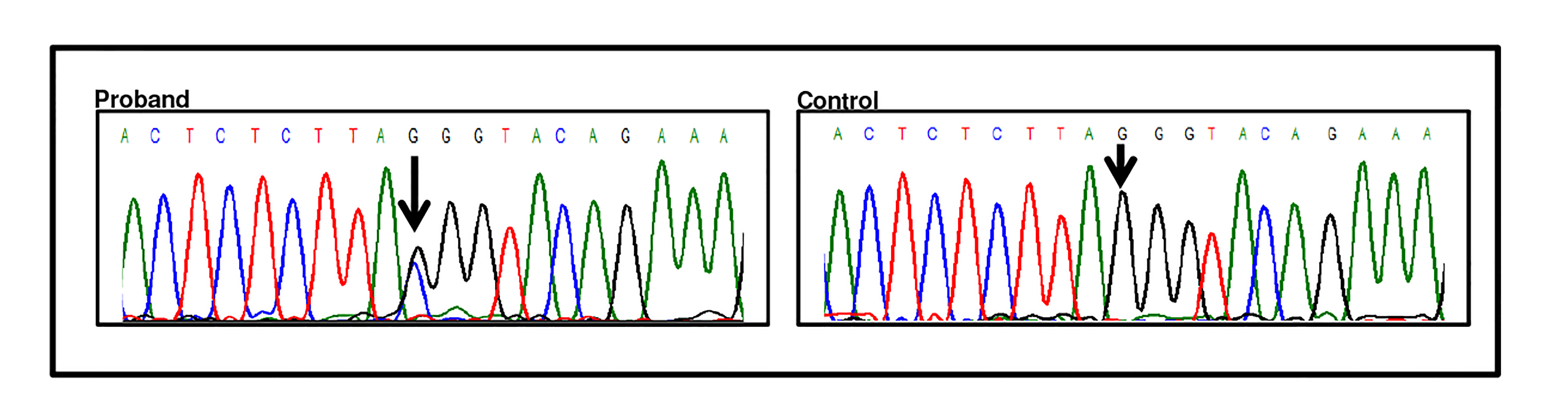

Supplement: S2 Fig — (TIF) [file pone.0121889.s002.tif]
