# Supplementary material for: Antithrombin III Deficiency in Indian Patients with Deep Vein Thrombosis: Identification of First India Based AT Variants Including a Novel Point Mutation (T280A) that Leads to Aggregation
Source: PLoS One. 2015 Mar 26;10(3):e0121889. doi: 10.1371/journal.pone.0121889 (PMC4374914; doi:10.1371/journal.pone.0121889)
Supplement: S1 Table — (DOCX) [file pone.0121889.s003.docx]

**Table S1.** Primer details used for PCR amplification of SERPINC1 gene

| **Amplicon** | **Primers** | **Sequences of oligonucleotidies**  **(5' ------- 3')** | **Localisation in**  **gene** | **Tm (^o^C)** | **Size (bp)** |
| --- | --- | --- | --- | --- | --- |
|  |  |  |  |  |  |
| Exon I | AT 1.5 | GAGAGGTGGCTCAGGCTTT | -190/-171 | 53.247 | 87 |
|  | AT 1.3 | AGCTCACCCCTCTTACCTTT | 108/127 | 51.780 |  |
| Exon II | AT 2.5 | TGTGGTGGGCAGTGGGGCTA | 2347/2366 | 57.930 | 367 |
|  | AT 2.3 | GGTGCTCCTAACAAGGTGGC | 2801/2819 | 55.880 |  |
| Exon III_a_ | PS 17C | AACTAGGCAGCCCACCAAA | 5260/5278 | 51.089 | 349 |
|  | PS 18B | TGCAACTCACCTTGAAGTCC | 5515/5534 | 51.780 |  |
| Exon III_b_ | E 3.5 | TGAATAGCACAGGTGAGTAG | 6388/6407 | 49.730 | 138 |
|  | E 3.3 | GCTGAAGAGCAAGAGGAAGT | 6600/6619 | 51.780 |  |
| Exon IV | E 4.5 | CCTCCTATGAATGTTTGTGT | 7334/7353 | 47.680 | 391 |
|  | E 4.3 | CTTTTGGTCAGACTACCTTG | 7806/7825 | 49.730 |  |
| Exon V | E 5.5 | TCTGTGGATTGAAGCCAACT | 9742/9761 | 49.730 | 66 |
|  | E 5.3 | CTGCTGTTCATGCATCTCCT | 9903/9922 | 51.780 |  |
| Exon VI | AT 6.5 | ATGAACGGCAGAGTGGCTAA | 13193/13212 | 51.780 | 260 |
|  | AT 6.3 | ATTTCAAATGCAGAGTCCAT | 13506/13525 | 45.630 |  |
| Promoter | AT PF | GCCTGAAGGTAGCAGCTTGT | -477/-458 | 53.830 | 300 |
|  | AT PR | CCCACACTCCCTCACTCTTC | -213/-193 | 55.880 |  |
| Intron 1 | AT I1F | ATCCGGGAAGAGAGCAAATGC | 436/457 | 54.357 | 1200 |
|  | AT I1R | CTCAAAACAGCAACAACAAAC | 1638/1658 | 48.500 |  |
| Intron 5 | AT I5F | TGGAGAGGAATTTGAAAG | 8037/8054 | 43.488 | 1080 |
|  | AT I5R | AACATCTTTCTTTCCAGTCTG | 9149/9169 | 48.500 |  |
| Intron 6 | AT I6F | GGGAGACTAGGGTGTTGA | 11539/11556 | 50.522 | 1200 |
|  | AT I6R | CAGATCTAGAGGGAAACACC | 12688/12706 | 51.780 |  |
| 3’UTR | AT 3‘UTRF | TTATCTTCATGGGCAGAGTAGC | 13377/13398 | 52.972 | 1000 |
|  | AT 3‘UTRR | ACACACAAGTAATAACATCCAC | 7825 | 49.245 |  |
