# Supplementary material for: Antithrombin III Deficiency in Indian Patients with Deep Vein Thrombosis: Identification of First India Based AT Variants Including a Novel Point Mutation (T280A) that Leads to Aggregation
Source: PLoS One. 2015 Mar 26;10(3):e0121889. doi: 10.1371/journal.pone.0121889 (PMC4374914; doi:10.1371/journal.pone.0121889)
Supplement: S2 Table — (DOCX) [file pone.0121889.s004.docx]

| **Polymorphism** | **Genotypic frequency** | **Anti-fXa (%)** | **Antigen (%)** |
| --- | --- | --- | --- |
| ***Pst*I** |  | | |
| **GG** | 32 (30.76%) | 80.5 ± 4.57 | 87.53 ± 4.01 |
| **GA** | 39 (37.5%) | 82.44 ± 5.82 | 91.97 ± 3.77 |
| **AA** | 33 (31.73) | 89.97 ± 4.22 | 95.39 ± 3.44 |
| **P value** |  | 0.4 | 0.36 |
| **rs2227589** |  | | |
| **GG** | 80 (76.9%) | 93.95 ± 2.65 | 98.33 ± 2.17 |
| **GA** | 19 (18.26) | 66.42 ± 5.37 | 78.11 ± 4.59 |
| **AA** | 5 (4.80) | 49.8 ± 13.29 | 60.8 ± 11.75 |
| **P value** |  | <0.0001 | <0.0001 |

**Table S2.** Genotype frequencies and their association with plasma AT activity and antigen levels in patients and healthy controls**.**
